# Embodied simulation in exposure-based therapies for posttraumatic stress disorder—a possible integration of cognitive behavioral theories, neuroscience, and psychoanalysis

**DOI:** 10.3402/ejpt.v6.29301

**Published:** 2015-11-20

**Authors:** Tuvia Peri, Mordechai Gofman, Shahar Tal, Rivka Tuval-Mashiach

**Affiliations:** Department of Psychology, Bar-Ilan University, Ramat Gan, Israel

**Keywords:** PTSD, exposure, mirror-neuron system, emotional regulation, psychotherapy integration

## Abstract

Exposure to the trauma memory is the common denominator of most evidence-based interventions for posttraumatic stress disorder (PTSD). Although exposure-based therapies aim to change associative learning networks and negative cognitions related to the trauma memory, emotional interactions between patient and therapist have not been thoroughly considered in past evaluations of exposure-based therapy. This work focuses on recent discoveries of the mirror-neuron system and the theory of embodied simulation (ES). These conceptualizations may add a new perspective to our understanding of change processes in exposure-based treatments for PTSD patients. It is proposed that during exposure to trauma memories, emotional responses of the patient are transferred to the therapist through ES and then mirrored back to the patient in a modulated way. This process helps to alleviate the patient's sense of loneliness and enhances his or her ability to exert control over painful, trauma-related emotional responses. ES processes may enhance the integration of clinical insights originating in psychoanalytic theories—such as holding, containment, projective identification, and emotional attunement—with cognitive behavioral theories of learning processes in the alleviation of painful emotional responses aroused by trauma memories. These processes are demonstrated through a clinical vignette from an exposure-based therapy with a trauma survivor. Possible clinical implications for the importance of face-to-face relationships during exposure-based therapy are discussed.

Exposure to the memory of the traumatic event is the common denominator of most evidence-based interventions for posttraumatic stress disorder (PTSD) and is included in the guidelines for the treatment of PTSD set by the International Society for Traumatic Stress Studies and by the Veterans Health Administration and Department of Defense (Foa, Keane, Friedman, & Cohen, 2009; VA/DoD, 2010). Yet, PTSD researchers and clinicians are divided with regard to the therapeutic process that underlies the curative effect of exposure. Cognitive behavioral therapy (CBT) approaches such as prolonged exposure claim that PTSD is mainly the result of impaired associative learning networks that cause intense fear responses to non-threatening stimuli (Foa & Kozak, 1986). Exposure to trauma memories and reminders in the safe environment of therapy supposedly changes this associative fear network or create new network associations that help patients to overcome their avoidance and prevent intense fear responses (Foa, 2011; Foa, Gillihan, & Bryant, 2013). Cognitive therapies such as Resick's Cognitive Processing Therapy (CPT) or Ehlers’ Cognitive Therapy stress the effects of excessive negative appraisals of the trauma and of distorted beliefs about the self and the world such as self-blame and overgeneralization. Through exposure to the trauma memory, cognitive therapy encourages patients to reevaluate and modify these erroneous cognitions (Ehlers et al., 2003; Ehlers & Clark, 2000; Resick, Nishith, Weaver, Astin, & Feuer, 2002; Resick & Schnicke, 1992). Narrative-based therapies such as narrative exposure therapy (NET), testimony therapy, and narrative reconstruction (NR) emphasize the impaired encoding of the trauma in the patient's memory system (Ehlers & Clark, 2000; Neuner, Schauer, Klaschik, Karunakara, & Elbert, 2004; Peri & Gofman, 2013; Schauer, Neuner, & Elbert, 2011; Weine, Kulenovic, Pavkovic, & Gibbons, 1998). These approaches claim that intrusions are the result of trauma memories that are neither integrated nor contextualized in the person's autobiographical memory. Exposure to the trauma memory through elaboration and reconstruction of the trauma narrative, orally or through writing, enhances integration and contextualization of the trauma memories and thus reduces uncontrolled recollections. Between typing the narrative, large portions of the session are dedicated to face-to-face discussion of the personal significance of the trauma.

Although therapeutic alliance has been found to be positively related to therapy outcome (Horvath, Del Re, Flückiger, & Symonds, 2011), and particularly patterns of change in alliance are important in prolonged exposure therapy (McLaughlin, Keller, Feeny, Youngstrom, & Zoellner, 2014), all of the above-mentioned approaches do not emphasize the significance of the patient–therapist interaction nor do they attribute a major role to the dynamic exchange of emotions between the patient and the therapist as a mechanism of change. In fact, a significant part of the exposure takes place outside of the therapy session during homework assignments conducted by the patient in solitude (Foa, Hembree, & Rothbaum, 2007), thus precluding direct interaction between patient and therapist.

Improvement in emotion regulation has been recognized as an important mediator of the effect of therapeutic alliance in exposure therapy for PTSD (Cloitre, Chase Stovall-McClough, Miranda, & Chemtob, 2004) and was found to be essential for effective psychotherapy with PTSD patients (Jerud, Zoellner, Pruitt, & Feeny, 2014). Various psychological and neuroscience theories have been suggested to understand the underlying mechanism by which emotion regulation is improved in psychotherapy. These include psychoanalytic theories like Kohut's theory about the role of self-objects in the development of emotional regulation (Kohut, 1984, 2010), Gergely's developmental theory of the role of contingency perception and marked mirroring during social interactions in early childhood (Gergely, 2007), and Schore's relational theory about the role of unconscious right brain communication between patient and therapist in the acquisition of better emotion regulation (Schore, 2014; Schore & Schore, 2008). All of the above theories describe processes occurring in the context of the patient–therapist relationship and point to the crucial role of that interaction during psychotherapy with PTSD patients.

In this work, we focus on the possible contribution of embodied simulation theory (ES; Gallese, 2005a) and embodied cognition (EC; Goldman & de Vignemont, 2009) to our understanding of the enhancement of emotion regulation in exposure-based treatments, suggesting an integration of current neuroscience and relational psychoanalytic formulations with classical learning theories in the understanding of the curative process. We propose that face-to-face interpersonal processes that take place during exposure therapy and which are based on the mirror-neuron system (MNS) make a specific and direct contribution to the attainment of increased control over fear responses and to enhanced regulation of negative emotions easily triggered in PTSD patients upon exposure to trauma reminders. This notion may have important implications for the development of clinical interventions for PTSD.

## Mirror neurons and ES

MNS was first discovered by Gallese and Rizzolatti in Macaque monkeys (Gallese, Fadiga, Fogassi, & Rizzolatti, 1996; Rizzolatti, Fadiga, Gallese, & Fogassi, 1996). They demonstrated that similar brain areas (premotor neurons and a sector of the posterior parietal cortex) are activated when a macaque monkey performs a motoric action as when the monkey is watching that action performed by another animal. It was soon demonstrated that not only the motor properties of the movement were mirrored, but their implied, implicit, goal and meaning were mirrored as well. Thus, for example, the same brain areas are activated in response to sounds characteristic of the movement or when the movement is not actually observed, but just implied (Gallese, 2009). A series of studies further demonstrated the existence of an MNS in humans which is involved in the imitation of simple movements and complex skills, and in the processing of action-related words and sentences (Gallese, 2009; Gallese, Eagle, & Migone, 2007). Other mirroring mechanisms were found to be involved with the human capacity to share emotions with others. Perceiving others feeling pain, disgust, or pleasure elicited similar responses in certain brain areas in the person experiencing these emotions and in the observer (Gallese et al., 2007; Goldman, 2011, 2012). Gallese (2009) concluded that these findings indicate that a human's capacity to empathize with the feelings of others is mediated by ES, that is, by the activation of the same neural circuits underpinning one's own emotional and sensory experiences (Gallese, 2005a, 2005b; Gallese, Keysers, & Rizzolatti, 2004).

The fact that mirroring phenomena have been demonstrated not only in relation to motor activities and emotional experience but also for the articulation and semantic aspects of language (Goldman & de Vignemont, 2009) adds to the relevance of ES to the field of psychotherapy, where verbal communication plays a central role. Reviewing recent findings of parallel bodily and neural responses in speaker and listener talking about emotional experiences, Gallese (2009) concluded: “It appears that even the apparently most explicit way of relating to others—that provided by linguistic expressions—is deeply rooted in intercorporeality” (p. 532).

Opponents of Gallese's extended theory of ES vary between those who minimize the role of the MNS to simple motor actions with almost no involvement of mirroring in empathy or mind reading (Decety, 2010; Gergely, 2007) to those who accept that mirroring is part of mind reading and empathy. The later claim that ES is involved in the social communication of basic or general emotional states, but that more detailed and complicated emotional communication involves simulation through cognitive processes described by theory of mind or cognition–cognition (CC) processes (for review, see Goldman, 2012; Goldman & de Vignemont, 2009), in which empathy is a deliberate cognitive process by which the observer has to “put himself in the shoes of the other” in order to understand and empathize with the observed. Thus, the observer develops a theory in his mind as to what is going in the mind of the other (i.e., the “other” cognition). For the purpose of this paper we may take a currently widely accepted position that both systems, the mirroring system based mainly on the MNS and the metalizing system that involves higher brain functions, work together in the process of emotion recognition (Spunt & Lieberman, 2012). In what follows we concentrate on the role of the MNS, which allows for fast and subconscious transmission of evolving emotional states of the patient to therapist through mirroring mechanisms and to the important role of this process in the enhancement of emotion regulation in exposure-based therapy for PTSD.

## ES in exposure-based therapies

**Fig. 1:**
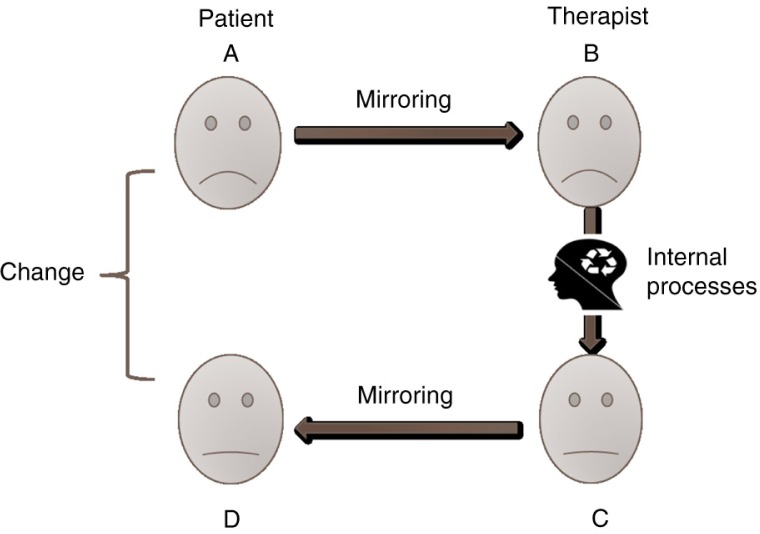
Mirroring as a mechanism of change in exposure therapy. Note: A—Patient recalls a painful memory accompanied with intense emotional pain expressed in his facial expressions. B—Therapist experiences viscerally the pain of the patient which is expressed in his face thus reassuring the patient and creating a “we-ness” feeling. C—Therapist activates emotional and cognitive regulation processes which moderate his emotional response. The moderated response is expressed in his facial expression. D—Modulated emotional responses are mirrored back by the patient who experiences his painful emotions in a modulated manner.

Exposure-based treatments are characterized by the patients repeating their traumatic memories while putting their experiences into words (Foa, 2011; Peri & Gofman, 2013; Schauer et al., 2011). This process commonly involves intense emotional responses as patients recall their trauma. We propose that, in addition to the extinction of conditioned fear responses and changes in cognitive schemata, the exchange of emotions between patient and therapist in exposure-based treatments plays an important role in the acquisition of emotional regulation (Fig. 1). We suggest that the therapist is not only listening to the patient's story, but is actually viscerally experiencing, through ES, the variety of emotions evoked in the patient while he or she describes the traumatic event. These emotions include not only fear but also a variety of other emotions such as shame, guilt, anger, or humiliation.

As mentioned above, current empirical findings regarding the role of the MNS are still limited to certain emotions like pain, disgust, or pleasure. Yet we may reasonably assume that mirroring is involved in other emotions and even in more complicated emotional responses that require further cognitive processing such as shame and guilt. It is plausible that the transmission of emotions is initiated through mirroring of a general emotional state represented in a bodily format which, in a second stage, is further elaborated through CC processes (Goldman, 2012). Alternatively, initial mirroring interacts with a second brain system which involves mainly frontal cognitive processes (Shamay-Tsoory, 2011). The fact that the therapist “feels” the patient's disclosed as well as dissociated emotions may contribute to the patient's recovery in several distinctive ways.

First, seeing the therapist's facial expressions and body gestures and hearing the therapist's voice all convey acknowledgement, validation, and identification which enhances a sense of “we-ness.” The patient feels that he or she is not alone in the pain and despair (Fig. 1B). Second, the therapist who correctly recognizes the patient's emotional states is able to accommodate his or her interventions to the patient's changing needs and to the patient's tolerance level at every stage of the therapy. The therapist can adjust the intensity and length of exposure to a level which the patient can bear, avoiding overwhelming responses such as dissociation or dropout. Third, the therapist's responses are not exact copies of the patient's. Gallese et al. (2007) proposed that the patient's mirrored emotions which are experienced by the therapist are moderated and regulated by the therapist and reflected back to the patient's MNS in an attenuated fashion (Fig. 1C). Patients can perceive and process a tempered image of their distressing emotions and thus improve their capacity to regulate and modulate their affective states. These modulated responses, and maybe even the modulation process itself, serve as models that the patients can internalize via the same process of ES, and may help them cope with former uncontrolled and unbearable emotions (Fig. 1D). The fact that not only fear is mirrored but probably a full array of negative emotions aroused by the trauma, may further help attenuate the patient's emotional distress through this bidirectional process of ES.

To incorporate our proposed mechanism of change into the common cognitive behavioral model, we claim that the modulation of emotional responses through ES processes is essential to the success of the processes of desensitization, extinction, and changes in underlying fear networks that lie at the heart of exposure-based therapy. It is important to emphasize that we do not claim that ES is solely responsible for these changes; however, we do propose that embodied communication of emotions plays a major role in the improvement of emotion regulation of fear responses together with other learning mechanisms. In the following clinical vignette, we demonstrate the above-mentioned processes. This vignette is from a therapy conducted as part of a study of NR therapy that was approved by the IRB of the university and by the Helsinki committee. Names and significant details were disguised to avoid any possible identification of the patient and therapist involved.

## A clinical vignette

Sara was a 36-year-old single woman who, for the first time, disclosed severe childhood abuse at the hands of her father from the age of 8. Her therapist was Lea, a clinical psychologist. She received NR therapy (Peri & Gofman, 2013)—a short-term, exposure-based, structured treatment module for intrusive memories. NR consists of a systematic and written chronological reconstruction of the full narrative of the trauma. The patient retells his or her experience while the therapist types the story on a computer. The therapist asks the patient to recall the full array of his or her actions, perceptions, cognitions, emotions, and sensations in a minute by minute manner. This recall and organization of the narrative is similar to other known interventions such as Ehlers and Clark's cognitive model for PTSD treatment (Ehlers & Clark, 2000; Ehlers et al., 2003) Resick's CPT (Resick et al., 2002), or NET (Neuner et al., 2004; Schauer et al., 2011); however, NR is conducted in a more detailed manner. Almost all of the sessions focus on the reconstruction rather than just being one element of treatment that combines other interventions like *in vivo* exposure and cognitive work. Reconstruction of the narrative includes invoking associated memories regarding past traumatic events or past interpersonal relationships that relate to the traumatic event. Special emphasis is given to the time period prior to and immediately after the traumatic event in order to examine the broader framework in which the trauma occurred, thus providing potential connections to the subjective emotional experience. Attention is also given to the developmental and environmental background of the patient, in an effort to enhance the integration of the trauma memory and to relate the traumatic event to the particular biography of the patient. Through this process the personal meaning of the trauma is elaborated upon and the trauma memory is actively integrated into the broader network of autobiographical memory. Between typing the narrative, significant parts of the session are devoted to face-to-face discussion of the traumatic event and its personal significance.

Sara described a severe episode of violent sexual abuse by her father when she was 8 years old. One day, she stayed home from school because she had a fever. Her father, who used to spend his days closed up in his room with his computer, asked Sara to come into his room. He locked the door and ordered his daughter to lie down on the bed. To the child's astonishment, her father handcuffed her to the bed while she was lying on her belly. Her father then undressed her and pushed an unidentified object inside her body over a long period of time. Despite her cries of pain this continued for a long time until she was released and sent out of the room with a warning not to tell anybody what happened. Furthermore, her father threatened her that even if she did tell anyone, no one would believe her story.

While telling her story, Sara oscillated between moments of intense pain, shame, and guilt to moments of detachment and apathy. For her therapist, the detailed narrative was difficult to comprehend. Reading the printed narrative in the sessions was extremely difficult for Lea. She struggled to hold back her tears while Sara related that during the night of the first assault she could not sleep and measured her temperature time and time again. She ended up taking a pill without permission to reduce her fever so that she would be able to go to school the next day and not be left again at home with her father. The image of the young girl awake all night praying for her fever to disappear was difficult to bear. Sara could see that her therapist's eyes were full of tears as she was struggling to contain the details of the story. This awareness served to reassure and comfort Sara who, for the first time in her life, was sharing her terrible experience. As Lea learned later, the abusive acts continued for some years after this event. Sara described her fear of her father's revenge in the event that she would reveal what had happened in the room on that day. This fear accompanied her for years to come.

The therapist moved back and forth between identifying with the pain expressed by Sara to some vague doubting of her patient's story. Gradually, the therapist could admit to herself that she was struggling between her identification with the painful experience Sara had endured and doubt that was emerging as to the veracity of the story. Lea felt that the story was “too much to be true.” The detached manner in which Sara related her story added to Lea's lingering sense that this might not have happened. At the same time, Lea felt uncomfortable expressing her inner doubts and had to conceal them for a while.

In the following session, Sara surprised her therapist by telling her about her own moments of inner doubt regarding the authenticity of her memories. She described feelings that echoed Lea's unspoken doubts regarding the truthfulness of such a terrible story while revealing more and more details regarding her father's aggressive and explosive behaviors toward her. These memories were supported by the facts related by Sara—the involvement of social workers in the family, police visits in their home, and the father's repeated refusal to enter therapy as was demanded by the authorities. The accumulation of these detailed memories clarified the painful reality of Sara's childhood experiences. It seemed that the therapist's growing confidence in the truth of Sara's memories reassured Sara and enabled her to further discuss her painful life history together with her feelings of confusion and questioning of the reality of her recollections.

## Vignette discussion

**Fig. 2:**
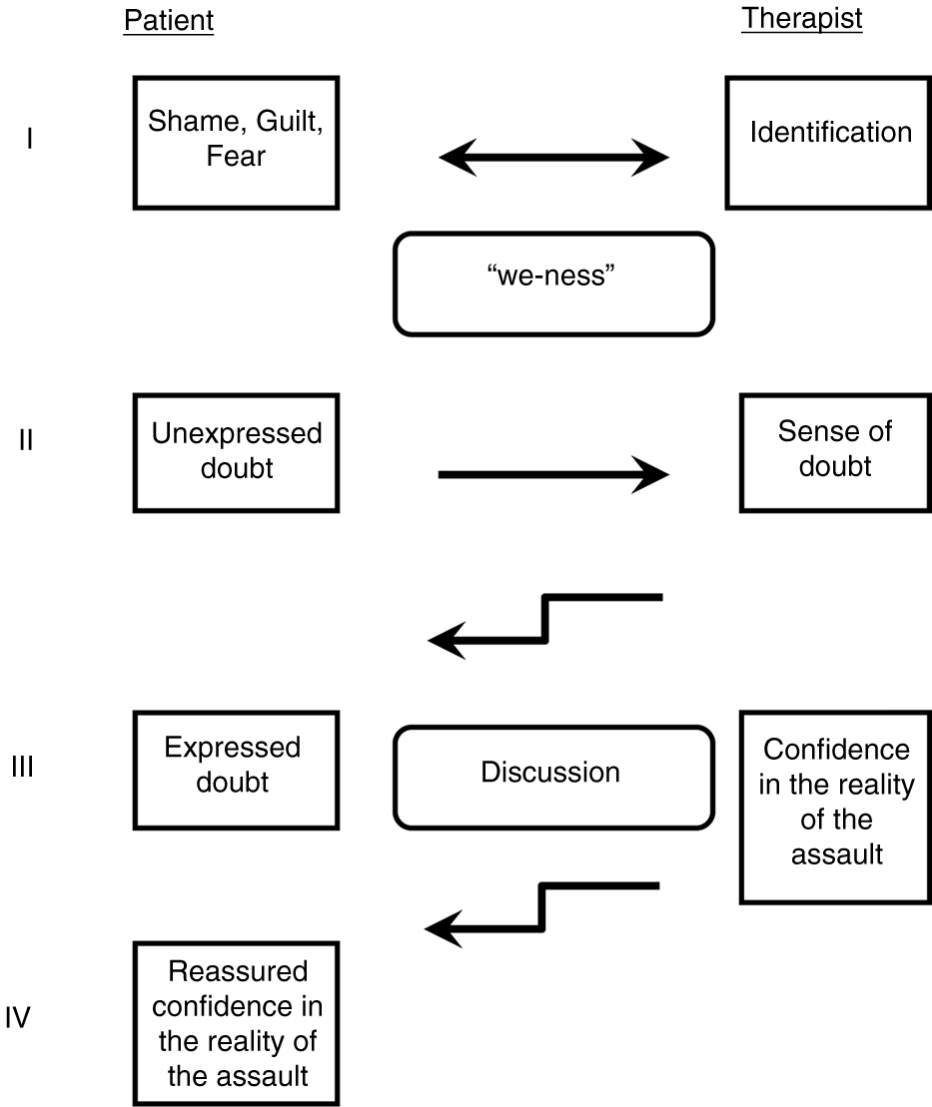
Mirroring processes between patient and therapist in PTSD therapy. Arrows indicate the direction of the transmission of emotion communication.

This vignette demonstrates the complex emotional exchanges that take place during exposure-based therapy. Figure 2 illustrates the elaborate transmission of emotions back and forth between patient and therapist that, according to our hypothesis, involves ES mirroring processes. In the case of Sara, the memory of a terrible childhood trauma and the combination of pain, fear, and guilt that accompanied the experience were mirrored and even felt by the therapist and thereby created a special “we-ness” atmosphere in the therapy (Fig. 2, stage I). Relating the trauma was followed by the appearance of unexpressed doubts in Sara's mind regarding the authenticity of her memories. Such doubts and memory disturbances are particularly common when the abuse involves betrayal by a parent (O'Rinn, Lishak, Muller, & Classen, 2013). These inner doubts are commonly reinforced by a message of disbelief from society and friends who can hardly believe that a parent could commit such cruel crimes. We propose that the doubts themselves or their related emotional cues regarding the authenticity of the memory were mirrored by the therapist and experienced by her (Fig. 2, stage II). The therapist's inner unexpressed doubts were then experienced by the patient (Fig. 2, stage III), which finally led to her explicit expression of her doubts, thus enabling an open discussion between patient and therapist. This discussion revealed additional detailed memories which convinced the therapist about the truthfulness of Sara's terrible experiences with her father. This growing confidence and belief of the therapist was transferred to the patient (Fig. 2, stage IV) and thereby reassured and enhanced her awareness that her therapist was with her in this journey through her painful past. We hypothesize that it was not only the pain, fear, humiliation, betrayal, and shame that were exchanged, but that the doubts regarding the truthfulness of the experience were shared as well. With the progress of the psychotherapy sessions, the painful conviction as to the authenticity of the memory was shared by both of them.

We propose that Sara suffered from a mixture of painful feelings regarding the traumatic experiences together with doubts regarding their reliability. These were intensified by her concern about her father's threat of revenge. The concurrent agonizing, painful doubts regarding the accuracy of her memories added to Sara's difficulty in disclosing her story. After the therapist's renewed conviction in the reality of the detailed and clear memory, the patient was reassured and was able to bear and express her oscillating thoughts between doubts and acceptance of the trauma.

As mentioned earlier, the therapist's mirrored emotions are not exact copies of the patient's ones. When these emotions are experienced by the therapist, they are modulated through complex interactions with the therapist's personal history and goals, through cognitive processing, and through emotional regulation processes. The final modulated response is the result of these complex interactions and is then experienced by the patient in a controlled and non-threatening fashion (Fig. 2, stage IV). We maintain that this process has an important role in any exposure-based therapy of PTSD.

## General discussion: toward an integrative perspective

In this work, we propose that the recent discoveries of the MNS and of ES theory may contribute an additional perspective to our understanding of the therapeutic mechanisms underlying exposure-based therapy for PTSD. We suggest that the emotional exchange between patient and therapist occurs in multiple stages. In the first stage, emotions such as fear, shame, and guilt experienced by the patient are mirrored by the therapist. These emotions are then modulated and reflected back to the patient in a controlled fashion. This process contributes to the improved regulation of the patient's painful emotions, thus enabling the change in emotional networks suggested by learning theories of PTSD.

Our suggested emphasis on the interpersonal exchange of emotions between patient and therapist corresponds with clinical insights and conceptualizations suggested by twentieth century psychoanalytic theories. Since its introduction, classical psychoanalysis has emphasized the role of detailed recollection of traumatic memories (Loewald, 1980) resembling the current conceptualization of exposure therapy. Loeweld (1980) wrote: “According to the description in this early paper [Freud, 1893] a cure occurred if the exciting event was brought to clear recollection, the accompanying affect aroused with the recollection, and if the patient related the event in as detailed a manner as possible and expressed his accompanying affects in words” (p. 40). Subsequent psychoanalytic clinicians and theorists emphasized interpersonal processes taking place between patient and therapist while working with memories of painful experiences. Kohut (1984) conceptualized *empathy* as not only a means of gaining knowledge of the patient's mind but as a primary vehicle of therapeutic action. According to Kohut, the patient's repeated experience of empathic understanding by the therapist serves to “repair” self-defects. Winnicott identified the *mirror-role* of the mother (Winnicott, 1971) in which the mother's facial expression reflects back the infant's emotions, thus encouraging emotional development. Winnicott (1971) wrote: “The mother is looking at the baby and what she looks like is related to what she sees there.” Stern, drawing upon both psychanalytic conceptualizations and developmental psychology termed what he called *emotional attunement* (Stern, 1985). According to Stern, in emotional attunement the caretaker takes the experience of emotional resonance and engages in a variation of the infant's own behavior, which the infant recognizes as corresponding to his or her own original feeling. All of the above are emotional processes in which the “therapist-mother” deeply understands the emotional state of the “patient-child,” reflects this emotional recognition to the patient, and responds to the patient's needs. Furthermore, psychoanalytic theories contend that through *projective identification* (Klein, 1946) the patient projects his or her unbearable emotions and thoughts “into” the therapist and, in turn, the therapist identifies with these emotions and experiences them as being his or her own (Mitchell, 1997; Ogden, 1979). Bion's concepts of *containment* and *reverie* (Bion, 1962) outline the therapist's role in helping the patient cope with his or her unbearable emotions. These emotions and thoughts are “digested” through the therapist's inner work, and then returned to the patient in a way he or she can sustain (Bion, 1962). Ogden (2004) describes this dynamic as the transformation of the unthinkable and even of the undreamable into regular emotional memories and thoughts. These processes resemble the proposed mirroring mechanisms we suggest are involved in common exposure-based therapies and are demonstrated in the emotional resonance of fear, shame, guilt, and doubt described in our case vignette.

The emotional communication between patient and therapist which takes place through ES has been used to elaborate upon the understanding of these psychoanalytic concepts in terms of their neurological basis (Gallese, 2009; Gallese et al., 2007). Galesse et al. suggested that the patient's emotions are expressed in his or her language, both in the words chosen and in its phonological characteristics, as well as in his or her bodily gestures and movements. The therapist who receives this information through the MNS is able to identify these emotions, to modulate them, and to reflect them back to the patient. As mentioned above, even opponents of the encompassing range of EC theory agree that current experimental findings support the mirroring of well-defined basic emotions such as pain, disgust, or pleasure. They propose that with regards to more complex emotional content such as guilt and shame, additional neuronal pathways are involved in the cognitive elaboration of these more complicated emotions as they are transmitted between people (Goldman, 2012; Shamay-Tsoory, 2011). Taken together, these theories describe well-defined brain mechanisms of emotional transmission between two people in social communication in general, and between patient and therapist during psychotherapy in particular.

Having elucidated psychoanalytic concepts through neuronal and cognitive functions opens the path to their integration into current, evidence-based CBT interventions. We propose that empathy, containment, emotional attunement, and projective identification are processes that may very well occur in exposure-based therapy and are important to the achievement of therapeutic change. Integration of psychoanalytic concepts with common CBT interventions may cultivate the latter with the experience and clinical insights gained through decades of psychoanalytic work with trauma survivors.

One major consequence of this proposed hypothesis is the reconfirmation of the importance of having a therapist present in face-to-face contact during exposure therapy. The therapist's presence not only reduces the feelings of loneliness and of being misunderstood, which are so common in PTSD patients, but also makes a direct contribution to the enhancement of the patient's ability to modulate negative emotional responses to the trauma memory. A possible conclusion of the above is that interventions including the presence of the therapist are more effective than similar interventions made by phone, email, or through internet-based self-help protocols without the presence of a therapist. Studies directly aimed at testing the effectiveness of internet-based interventions have shown that a positive therapeutic alliance may be established and that they may have considerable positive effects on posttraumatic symptomatology (Shaili, 2011; Sucala et al., 2012) and may even be preferred by some PTSD patients (Spence et al., 2011). Yet even studies that found positive effects of internet-based interventions for PTSD suffered from high dropout rates, small sample sizes, and short-term follow-ups, thus limiting our ability to reach definitive conclusions (Brief et al., 2013; Shaili, 2011). A possible research method for a closer test of our hypothesis may be to directly measure the synchronization of patient and therapist's emotional flow during psychotherapy sessions; and the relationship between synchronization level and outcome in exposure-based psychotherapy. Emotional fluctuations may be evaluated through different psychophysiological measures such as heart rate or skin conductance responses (for review, see, Marci & Riess, 2009) or through vocal measures, such as changes in voice prosody (Moneta, Penna, Loyola, Buchheim, & Kächele, 2008) or vocal acoustics (Rochman, Diamond, & Amir, 2008).

Although the present discussion of the suggested integrative theory focuses on exposure-based therapies, the model may be involved in almost any face-to-face interaction in other psychotherapies. Yet, this generalization of the theory requires further research. This work suggests that the therapist has a somewhat different role in exposure-based therapies than previously considered in cognitive behavioral models. The therapist is more than just the facilitator of exposure in a safe and calm environment. According to our hypothesis, exposure is also an interpersonal experience in which two complementary mirroring-based processes are taking places: 1) emotions are shared thus creating a sense of “we-ness” in the therapy; and 2) emotions are modulated in a way that simultaneously reduces fear as well as enhances regulation of a wider range of feelings. The interpersonal aspects of exposure therapy, as discussed above, may require an additional skill set for the therapist to further enhance the practice of exposure in psychotherapy for PTSD.
